# Supplementary material for: From work stress to disease: A computational model
Source: PLoS One. 2022 Feb 16;17(2):e0263966. doi: 10.1371/journal.pone.0263966 (PMC8849534; doi:10.1371/journal.pone.0263966)
Supplement: S1 Appendix — (DOCX) [file pone.0263966.s001.docx]

**Supplementary figures from “From Work Stress to Disease: A Computational Model” Remco Benthem de Grave, Fred Hasselman, & Erik Bijleveld**

**Exponential Distribution for Time Points of Night Impulses**

|   *Figure*, density plot of random draws from the exponential distribution, used to determine the time point of the night impulses. The plot illustrates the likelihood that a particular random draw will have a certain time value, a higher density reflecting a higher likelihood that a night impulse will happen at that particular timepoint. For use in the simulation, the drawn timepoint of the night impulse was rounded to the nearest time sample of the simulation. |
| --- |

**Distributions of Individual Averages**

|   *Figure*, distributions of individual averages of night impulses and work impulses of 10,000 simulated people. The plot illustrates the likelihood that a certain simulated individual will have a particular average number of work and night impulses per day. |
| --- |

**Cortisol time courses of Scenario I and II**

|   *Figure*. Simulated cortisol time courses since awakening as obtained from simulating Scenario I, with no correlation between work and night impulses (a) and Scenario II, with correlation between work and night impulses (b). Inspection of both plots suggests that the simulated cortisol time courses are extremely similar in both scenarios. |
| --- |

|   *Figure*. Cortisol time courses throughout the night from simulating Scenario I, with no correlation between work and night impulses (a) and Scenario II, with correlation between work and night impulses (b). Also here, inspection of both plots suggests that the simulated cortisol time courses are extremely similar in both scenarios. |
| --- |

**Allostatic Load vs. Work Impulse Frequency**

*
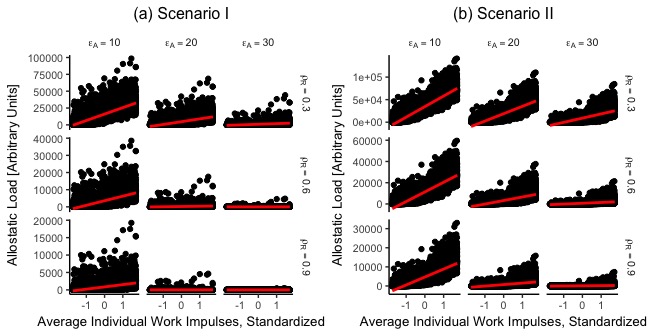
Figure.* Simulated allostatic load against the average frequency of work impulses (standardized) for Scenario I, no correlation between work and night impulses (a) and Scenario II, correlation between work and night impulses (b). Different subplots within each panel represent the different parameter combinations that we examined. The plot depicts the distribution of the developed allostatic load of the simulated individuals. Thus, this plot is closely related to, and consistent with, the odds plot of relative disease risk (Figure 9in the manuscript).

**Disease and Allostatic Load Ratios**

Table

For each tested parameter combination in Scenario I (work impulses and night impulses uncorrelated), the table shows the percentage of simulated individuals developing disease at any time during the 200 days of the simulation. This table illustrates that the predicted relation between work stressors and disease (i.e., Relative Risk > 1) is found often at unrealistically high percentages of simulated people getting diseased (see main text).

|  | Parameter Values | | |  | |  |  |
| --- | --- | --- | --- | --- | --- | --- | --- |
| Simulation | $\rho_{E}$ | $\varepsilon_{A}$ | $\varepsilon_{D}$ | | Diseased (%) | | Relative Risk>1^a^ |
| 1 | 0.3 | 10 | 100 | | 96.1 | | yes |
| 2 | 0.3 | 10 | 400 | | 93.0 | | yes |
| 3 | 0.3 | 10 | 1000 | | 89.3 | | yes |
| 4 | 0.3 | 20 | 100 | | 73.4 | | yes |
| 5 | 0.3 | 20 | 400 | | 64.6 | | yes |
| 6 | 0.3 | 20 | 1000 | | 56.2 | | yes |
| 7 | 0.3 | 30 | 100 | | 40.0 | | yes |
| 8 | 0.3 | 30 | 400 | | 30.0 | | yes |
| 9 | 0.3 | 30 | 1000 | | 21.3 | | yes |
| 10 | 0.6 | 10 | 100 | | 81.7 | | yes |
| 11 | 0.6 | 10 | 400 | | 72.0 | | yes |
| 12 | 0.6 | 10 | 1000 | | 61.5 | | yes |
| 13 | 0.6 | 20 | 100 | | 23.4 | | yes |
| 14 | 0.6 | 20 | 400 | | 11.7 | | yes |
| 15 | 0.6 | 20 | 1000 | | 4.8 | | yes |
| 16 | 0.6 | 30 | 100 | | 1.1 | | no |
| 17 | 0.6 | 30 | 400 | | 0.5 | | no |
| 18 | 0.6 | 30 | 1000 | | 0.3 | | no |
| 19 | 0.9 | 10 | 100 | | 57.2 | | yes |
| 20 | 0.9 | 10 | 400 | | 41.6 | | yes |
| 21 | 0.9 | 10 | 1000 | | 27.1 | | yes |
| 22 | 0.9 | 20 | 100 | | 2.3 | | no |
| 23 | 0.9 | 20 | 400 | | 0.9 | | no |
| 24 | 0.9 | 20 | 1000 | | 0.5 | | no |
| 25 | 0.9 | 30 | 100 | | 0.1 | | no |
| 26 | 0.9 | 30 | 400 | | 0.1 | | no |
| 27 | 0.9 | 30 | 1000 | | 0.0 | | no |

^a^This column describes whether one SD increase in work impulses predicted a significantly higher risk of becoming diseased. We tested the 95% confidence interval of the odds ratio (OR) against the condition of no noticeable difference (an OR of 1).

**Relative Risks for Worktime Configurations Under Various Parameter Combinations**

| ****  *Figure*. For the various tested parameter combinations, predictions of relative disease risk from worktime configurations, compared to the standard week (always configuration #1 for the specific parameter combination). The figure illustrates that, independently of the parameter values used, the pattern is always the same. That is, no difference was found between the standard work week (configuration #1, working Mon-Fri, 8h daily) and configuration #2 (Mon–Tue and Thu–Sat, 8h daily). An increase in disease risk was found for configuration #3 (Mon–Tue and Thu–Fri, 10h daily) and #4 (Mon–Tue and Thu, 13h20m daily), both representing a workweek with working hours concentrated in fewer working days. A decrease in disease risk was found for configuration #5 (Mon-Sat, 6h40m daily) and #6 (Mon-Sat, 5h43m daily), both representing a workweek with working hours spread out over a higher number of working days. |
| --- |

**Growth Curves of Allostatic Load of People Becoming Diseased**

| ****  *Figure*. For different parameter combinations, growth curves of allostatic load are shown for 100 simulated people that had become diseased at the end of the simulation, after 200 simulated days. Each colour represents a different simulated person. The figure illustrates how allostatic load developed over time in these individuals, suggesting that process happens at a constant, linear rate. |
| --- |
