# Supplementary material for: From work stress to disease: A computational model
Source: PLoS One. 2022 Feb 16;17(2):e0263966. doi: 10.1371/journal.pone.0263966 (PMC8849534; doi:10.1371/journal.pone.0263966)
Supplement: S1 Fig — (PDF) [file pone.0263966.s002.pdf]

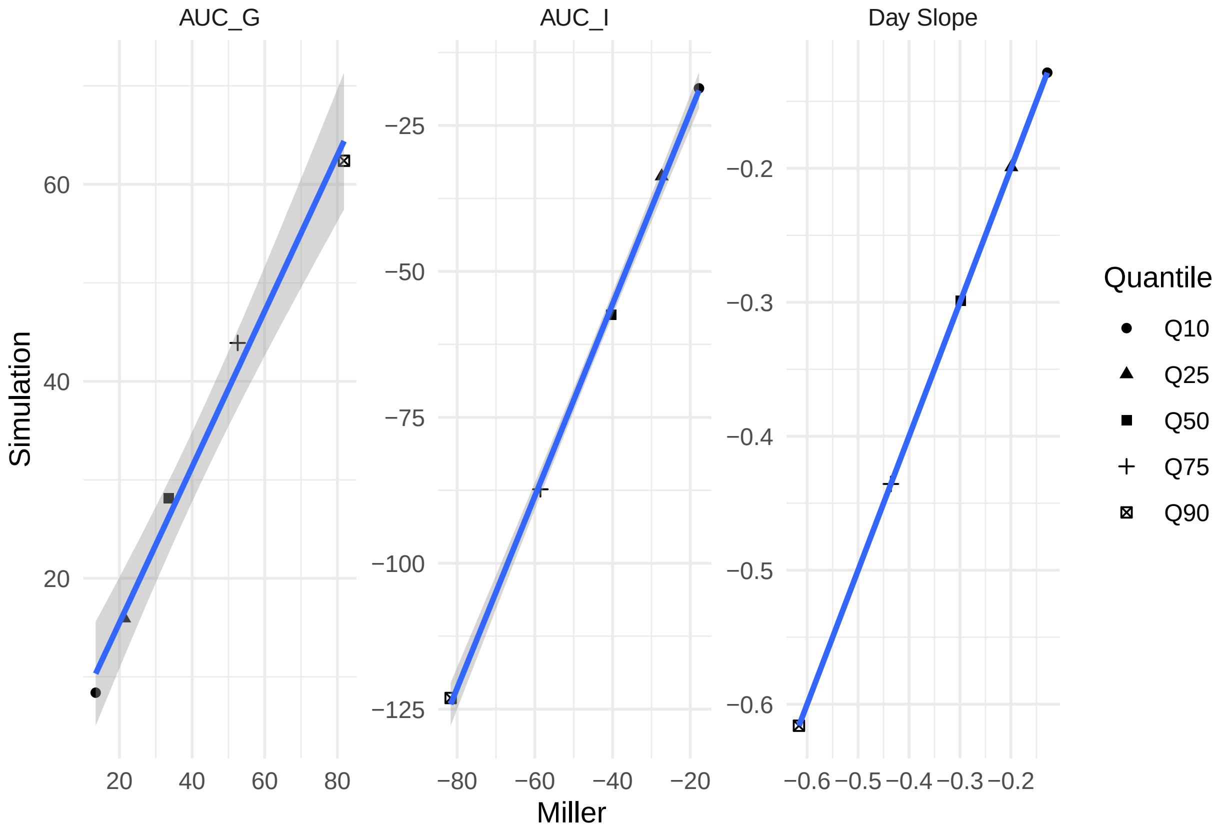

Comparing aggregated data presented in Miller et al. [1] and aggregation of our simulation by quantile (10%, 25%, 50%, 75% and 90% quantiles) on AUC_G, AUC_I and an approximation of wake-to-bed slopes #. In the case of a perfect replication of the data from Miller by our simulations, we expect indices to be proportional between the quantiles. In other words, a straight line connecting the indices at the quantiles is suggestive of a perfect fit.

Wake-to-bed slopes can only be approximated, as only the wake time is synchronized and individual bed times are unknown. We have used the aggregated samples at 15h after wake as the bed time.

(1) Miller R, Stalder T, Jarczok M, Almeida DM, Badrick E, Bartels M, et al. The CIRCORT database: Reference ranges and seasonal changes in diurnal salivary cortisol derived from a meta-dataset comprised of 15 field studies. Psychoneuroendocrinology. 2016;73. doi:10.1016/j.psyneuen.2016.07.201.
